# Treatment and Prevention of HPV-Associated Skin Tumors by HPV Vaccination

**DOI:** 10.3390/vaccines12121439

**Published:** 2024-12-20

**Authors:** Thomas Meyer, Eggert Stockfleth

**Affiliations:** Department of Dermatology, St. Josef Hospital, Ruhr University Bochum, Gudrunstrasse 56, 44791 Bochum, Germany; eggert.stockfleth@klinikum-bochum.de

**Keywords:** papillomavirus, skin cancer, genital warts, cutaneous warts, oncogene, skin microbiome, commensal, immune surveillance, virus-like particle

## Abstract

HPV-associated dermatological diseases include benign lesions like cutaneous warts and external genital warts. In addition, HPV infection is associated with the development of epithelial skin cancers, in particular cutaneous squamous cell carcinoma (cSCC). In contrast to anogenital and oropharyngeal cancers caused by mucosal HPV types of genus alpha papillomavirus, cSCC-associated HPV types belong to the genus beta papillomavirus. Currently available HPV vaccines that target mucosal HPV types associated with anogenital cancer and genital warts are type-specific and provide no cross-protection against beta HPV. When implementing vaccination to beta HPV to prevent skin tumors, it must be considered that acquisition of these HPV types occurs early in childhood and that the risk for cSCC increases with growing age and decreasing immune surveillance. Thus, individuals considered for beta HPV vaccination usually have pre-existing infection and are largely immunocompromised. On the other hand, worldwide increasing incidence rates of epithelial skin cancer reflect an urgent need for skin cancer prevention measures. Based on the pathogenic involvement of beta HPV, vaccination may represent a promising prevention strategy. Indeed, various procedures of prophylactic and therapeutic vaccination have been developed, and some of them have shown efficiency in animal models. Thus far, however, none of these vaccine candidates has been approved for application in humans.

## 1. Introduction

HPV-associated dermatological diseases include benign lesions like cutaneous warts and external genital warts. In addition, HPV infection is associated with the development of epithelial skin cancers, in particular cutaneous squamous cell carcinoma (cSCC). In general, these skin lesions are associated with specific subsets of HPV types that are different from mucosal HPVs causing cancer of the cervix, vagina, vulva, anus, rectum, penis, and oropharynx [1,2]. Due to their oncogenic potential, mucosal cancer-associated HPV types were named high-risk HPVs and include HPV 16 and 18, which are detected most frequently in anogenital and oropharyngeal cancers, as well as another 12 less prevalent HPV types (31, 33, 35, 39, 45, 51, 52, 56, 58, 59, 66, and 68) [3,4]. Immunization against high-risk HPVs has been shown to be very effective in preventing HPV infection and subsequent cervical dysplasia and carcinoma [5,6]. Currently available vaccines are based on virus-like particles (VLPs) that self-assemble from the major capsid protein L1 and induce high levels of neutralizing antibodies that prevent HPV de novo infection [7]. Whereas the preventive efficacy of these HPV vaccines is widely accepted, it is controversial whether vaccination with L1-based VLPs may also affect established or active HPV infection. Furthermore, antibodies induced by L1-VLP vaccines are largely type-specific, with limited cross-protection against other HPV types. Thus, when expanding HPV vaccination to skin tumors, it must be considered that other HPV types are involved in skin tumors and acquisition of these HPV types occurs early in childhood by transfer through common household contacts, in contrast to genital HPVs, which are transmitted primarily by sexual contacts [8].

Epithelial skin cancers include basal cell carcinoma (BCC) and cutaneous squamous cell carcinoma (cSCC). Together they represent the vast majority (>99%) of nonmelanoma skin cancers (NMSC), whereby BCC are four times more frequent than cSCC (80% vs. 20% of all NMSC). The incidence of these cancers is much higher than that of any other skin cancer. Several studies have shown increasing incidence rates of epithelial skin cancer in different countries, including the United States, United Kingdom, Denmark, and Germany [9]. According to the Schleswig-Holstein cancer registry in Germany, age standardized incidence rates of cSCC increased from 39.0 to 68.6 per 100,000 person-years in men and from 19.6 to 36.1 per 100,000 person-years in women between 1999 and 2020. Incidence rates continuously increased for women and men until 2013 and 2015, respectively. The extrapolation of these data for the next 20 years predicts an ongoing increase of age standardized incidence rates, up to 115 per 100,000 person-years in males and 63 per 100,000 person-years in females [10]. Thus, an increase of cSCC will be expected, especially in people aged >60 years with men being more frequently affected than women, representing a significant burden for the health care system and reflecting the need for preventive measures [11].

Next to individual factors like age, pigmentation and genetic predisposition (Xeroderma pigmentosum, basal cell nevus syndrome, epidermodysplasia verruciformis) [12,13,14], UV exposure, immune status, and HPV infection represent important environmental risk factors for development of epithelial skin cancers. The most important risk factor for the development of skin cancer is chronic exposure to ultraviolet radiation [15]. Although DNA damages caused by UV and high-intensity light may be removed by various DNA repair systems, they may fail over time. Lifetime sun exposure and the number of painful sunburns before the age of 20 years were associated with an increased risk of cSCC, and to a lesser degree with nodal and superficial BCC [16].

The importance of immune surveillance (i.e., immunological functions to control and remove abnormal and malignant cells) for development and progression of cSCC is reflected by the higher risk of cSCC development in organ transplant recipients (OTRs) receiving iatrogenic immunosuppression, which is 65–250 times higher than in general population [17,18,19,20,21]. In addition to UV radiation and immunosuppression, infection with specific cutaneous HPV types may represent another exogenous risk factor for NMSC, described in more detail in the next chapters. Given the association of HPV with development of epithelial skin cancer vaccination against involved HPV types may represent a promising prevention strategy. The number of patients who may benefit from such a vaccine is likely to accumulate in the future, as the frequency of these skin cancers will increase with higher life spans, prolonged exposure to UV-light, and impairment of immune functions.

In this review we aim to compile vaccination strategies to prevent HPV-associated skin tumors. They differ from the currently available vaccines for mucosal alpha HPVs, as skin-tumor-associated beta HPVs have different biological and oncogenic activities. Therefore, we start with a brief comparison of potential carcinogenic mechanisms of alpha and beta HPVs.

## 2. Human Papillomaviruses (HPV)

The family of *Papillomaviridae* includes a large number of double-stranded, non-enveloped, small DNA viruses that are widely prevalent in humans and numerous animal species [22]. Individual virus types differ with respect to their biological properties, like host specificity, tissue tropism (mucosal or keratinized epithelial cells), and oncogenic potential. In humans, 231 HPV types have been fully sequenced and another 230 partial sequences were identified, probably representing additional HPV types (http://pave.niaid.nih.gov, http://www.hpvcenter.se; searched on 11 December 2024).

HPV divide into five different genera within the family of *Papillomaviridae*: *alpha-*, *beta-*, *gamma-*, *mu-*, and *nu-papillomavirus* [23]. All mucosal HPV types belong to the genus *alpha-papillomavirus*. Some of them were classified as low risk and high-risk HPV types, based on their relative frequency in cervical cancers [24]. Most cutaneous HPV types infecting keratinized epithelia belong to the genera *beta-* and *gamma-papillomavirus*. A small number of cutaneous HPVs were classified as *mu-* and *nu-papillomavirus* (http://pave.niaid.nih.gov). These HPV types are usually associated with benign epithelial lesions. Gamma-, mu-, and nu-HPVs may cause common or plantar warts; beta HPV infection is mostly subclinical or may present as flat warts [25]. However, increasing evidence suggests that at least a subset of beta HPV is involved as a co-carcinogen in addition to UV-radiation in the development of cSCC [26,27].

Many new HPV types were detected by amplification-based detection methods using consensus primers directed against conserved regions of the HPV genome; however, the spectrum of detected HPV types is limited by the degree of homology of the primers used. In contrast, metagenomic analyses using whole genome sequencing principally allows detection of all HPV sequences. Using these technologies, HPV sequences were detected in condylomata acuminata that tested negative for HPV by traditional PCR methods [28]. By comparing sequencing data from 748 samples of 103 healthy human subjects of the NIH Human Microbiome Project (HMP) with HPV sequences available in the Virus Episteme (PaVE) database (http://pave.niaid.nih.gov) and the International HPV Reference Center (http://www.hpvcenter.se), 109 known HPV types and many unclassified types were detected [29]. HMP samples were from four different organs and HPV prevalence was highest in skin (61.3%), followed by vagina (41.5%), mouth (30%), and gut (17.3%) with more than one HPV type in most of the samples. These findings indicate a more complex HPV community in healthy humans than previously assumed in studies using HPV detection methods selective for specific HPV types. The presence of multiple HPV types in healthy subjects may indicate a physiological role of HPV. As a possible mechanism, non-oncogenic HPV types in mixed infection may reduce the cancer risk by inhibiting oncogenic viruses through cross-immunity or viral interference [30].

## 3. Mechanisms of HPV-Induced Cancers

### 3.1. Alpha HPV

Upon establishment of the causal relationship of genital HPV infection and cervical cancer in 1983 [1], it has been shown that these HPV infections are frequently cleared immunologically in healthy women, but persistent infection with high-risk mucosal HPV types is associated with progression to cervical cancer [1]. The HPV genome consists of three regions: the early region containing genes E1, E2, E4, E5, E6, and E7, required for early stages of infection, replication, cell cycle control, and oncogenesis; the late region, containing capsid proteins L1 and L2; and a non-coding long control region involved in control of virus replication and transcription [31]. E6 and E7 are the main viral oncoproteins. Their most important oncogenic activity is the inactivation of cellular tumor suppressor proteins p53 and pRB, respectively, resulting in uncontrolled cell proliferation by dysregulation of cell cycle control and apoptosis [32]. In most cases of cervical cancer, the HPV genome is integrated into the host genome [33], which results in disruption of E2 and activation of E6 and E7 gene expression, as E2 is a negative regulator of oncogene expression [34]. In addition, disruption of E2 causes blocking of viral replication, which is depending on interaction of intact E1 and E2 proteins [35]. Furthermore, interaction of HPV16 E2 with DNA topoisomerase 2-binding protein 1 (TOPBP1) activates the DNA damage response (DDR) during mitosis to promote TOPBP1 interaction with mitotic chromatin, and thus segregation of the viral genome [36]. Next to the inactivation of p53 and pRB, a number of other activities of E6 and E7 proteins were identified that contribute to the oncogenic potential of high-risk alpha HPVs [1,31,37]. These activities result in uncontrolled cell proliferation, inhibition of cell differentiation, apoptosis, and DNA repair, as well as in immortalization, cell invasion, and evasion of immune reactions, which are all features representing so-called hallmarks of cancer.

While E6 and E7 provide the primary transforming activities of HR-HPV, the E5 protein may augment their functions and contribute to carcinogenesis. The most important oncogenic functions described for E5 include activation of epidermal growth factor receptor (EGF-R) and downstream mitogen-activated protein (MAP) kinase pathways or the PI3K-Akt pathway, as well as suppression of the cyclin-dependent kinase inhibitor p21, which may stimulate cell proliferation and angiogenesis. Furthermore, E5 may contribute to malignant progression by impairing apoptosis [38] and by inducing aberrant expression of mesenchymal fibroblast growth factor receptor (FGFR) 2c, epithelial mesenchymal transition, and cell invasion [39]. As the E5 gene is frequently deleted during integration of the HPV genome, the role in carcinogenesis seems to be limited to early stages of malignant progression [1].

### 3.2. Beta HPV

High-risk mucosal HPVs may be considered pathogenic tumor viruses that cause symptomatic infections. In contrast, beta HPVs usually do not cause symptoms and are ubiquitously present in humans, thus rather representing commensal than pathogenic viruses. In fact, the ubiquitous prevalence of beta HPVs in the general healthy population [40,41] raise doubts about a causal involvement in cSCC development. Furthermore, beta HPVs lack the E5 gene and do not integrate into the host cellular genome [41,42,43]. Thus, E5 seems to have given a carcinogenetic boost to alpha HPVs compared to beta HPVs. Beta HPVs were detected in both normal skin and skin tumors and are more prevalent in precancerous lesions than in skin cancers (keratinocyte cancers, KC). The viral load and viral gene expression is also higher in precancerous lesions than in KC [44,45].

However, OTRs, who require immunosuppressive therapy, have an increased risk for KC, in particular cSCC, and also have an increased risk for beta HPV infection [17]. Whereas beta HPVs were detected in up to 90% of healthy skin of both immunocompetent and immunocompromised patients, beta HPV DNA is detected in 80% of cSCC of immunosuppressed patients, compared to 40% of cSCC of immunocompetent patients [46]. Furthermore, in OTRs, beta HPV detection rates in hair follicles and antibodies against beta HPV correlate with cSCC development [26,47], indicating that immunosuppression leads to higher beta HPV loads and increased oncogenic activity, which in turn increases the risk of cSCC development [48].

Further evidence for involvement of beta HPV in skin carcinogenesis is provided by several in vivo studies using *Mastomys natalensis papillomavirus* (MnPV) and transgenic mice models. When *Mastomys coucha* animals were infected with MnPV, most of them developed cSCC when exposed to UV radiation, whereas few animals not infected with MnPV but exposed to the same UV dose, as well as non-UV-exposed but MnPV-infected animals, develop cSCC [49]. In other studies with transgenic mice containing the HPV8 early region under control of the keratin 14 promoter to limit expression to the basal layer, spontaneous development of cSCC and other cutaneous lesions was shown [50]. Integration of E6 and E7 of HPV38 and exposure to chronic UV radiation results in development of actinic keratosis (AK)-like lesions and cSCC, in contrast to wild-type mice that do not develop skin tumors when exposed to the same UV dose [51]. Knocking down HPV8 E6 expression with gene specific siRNA resulted in delayed or absent development of papillomas [52], indicating HPV8 gene expression seems to be required for tumor development. In addition, silencing of E6 and E7 expression in transgenic mice with already existing cSCC did not affect the tumor phenotype, indicating a role of HPV in the early steps of skin carcinogenesis [53].

In contrast to alpha HPVs, E6 proteins of beta HPVs do not bind to the ubiquitin ligase E6AP. Instead, beta HPV E6 proteins interact with the Mastermind-like transcriptional coactivator 1 (MAML1) to inhibit signaling of NOTCH, a tumor suppressor in squamous epithelial cells [54]. In addition, beta HPV E6 induces degradation of acyltransferase p300, resulting in reduced expression of breast cancer (BRCA) 1 and BRCA 2, and thus impairing double-strand DNA repair (DSR) mechanisms [55].

On the other hand, E7 of both alpha and beta HPV bind to pRB, although binding of at least some beta HPV types is weaker than for alpha HPVs [56]. A number of other mechanisms were described for beta HPVs that interfere with cellular functions, like cell cycle control, apoptosis, DNA repair, keratinocyte differentiation, and immune functions, and thus may be considered tumor-promoting activities [26,37,57]. Many of these mechanisms vary among beta HPVs with respect to potency, or have only been shown in subsets of beta HPVs [58].

Of importance, however, inhibition of Notch signaling and p300 results in cell proliferation despite impaired double-strand DNA repair (DSR) and inhibition of apoptosis that may allow accumulation of genomic mutations required to progress to cancer. This points towards a role of beta HPVs in early stages of cSCC development, with viral activities involved in the initiation of cell transformation that are no longer required for tumor maintenance, consistent with a “hit and run“ mechanism of beta HPV plus UV radiation in skin cancer development [26].

On the other hand, a recent study of Strickley et al. showed that beta HPVs, as part of the human skin virome, may have beneficial effects for the host. Beta HPVs induce T-cell immunity that is also directed against virus-infected dysplastic keratinocytes, and thus may protect against cSCC development [59]. In the context of immunosuppression or immunosenescence, however, reduced T-cell mediated immunity may fail to eliminate HPV-infected keratinocytes and facilitate skin carcinogenesis in cooperation with UV radiation. This hypothesis is supported by experiments using the MmuPV1 mouse papillomavirus back skin model, where virus-infected mice develop fewer skin tumors than non-infected mice after exposure to chemical carcinogens or UV radiation. The protective effect was mediated by CD8+ tissue resident memory T cells (T_RM_). Moreover, beta HPV-reactive T_RM_ (anti E7) were also detected in normal human skin, indicating adaptive immunity against commensal HPVs in healthy adults [59]. These epithelial T_RM_ cells may protect against beta HPV-induced wart development, but also control beta HPV-infected cells undergoing UV-induced transformation, and thus suppress formation of cSCC. Under immunosuppression, increasing viral burden (increasing viral load and/or multiplicity of HPV types), possibly due to reduced T_RM_ mediated immunity, becomes pro-carcinogenic in cooperation with UV radiation. The impairment of T_RM_-mediated protection against HPV-infected and -transformed keratinocytes may result in escape of dysplastic cells, in particular those that are not HPV-infected, providing an explanation for the fact that HPV DNA is less frequently detected in cancer cells of cSCC.

Whatever the role of beta HPV is in human epithelial cancer, being a constituent of the human skin microbiome that promotes development of local immune functions, or being an oncogenic factor for epithelial cancer, or both, the enhancement of immune functions directed against virus-infected keratinocytes, which potentially may become dysplastic either by viral activities or other factors, would be an obvious approach to prevent the development of epithelial cancers. Strengthening of immune reactions against beta HPVs may be achieved by prophylactic and therapeutic vaccination against these HPV types. This is of particular importance in immunosuppressed patients that have a much higher risk of tumor development, and ideally should by applied prior to immunosuppression.

## 4. Therapeutic Interventions to Inhibit HPV-Associated Cutaneous Diseases

**Figure 1:**
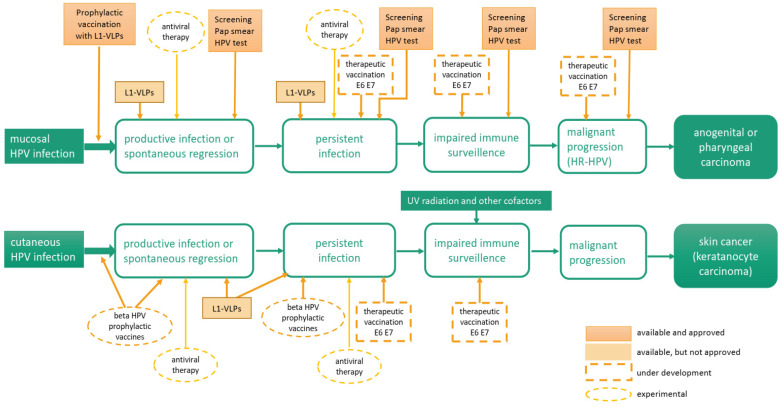
Interventions available and under development for mucosal and cutaneous HPV infections. Prophylactic vaccination with licensed L1-VLPs and screening by cytology and HPV tests are established methods to prevent or monitor mucosal HPV infection. Although not approved, L1-VLPs may also be used to treat pre-existing infection. Prophylactic vaccination targeting beta HPVs and therapeutic vaccination against mucosal and cutaneous HPVs are still under development. Antiviral compounds against HPV proteins are presently not on hand and the development is still in an experimental stage.

Any interaction with the expression or activity of HPV oncogenes may be exploited for therapeutic intervention to inhibit HPV carcinogenesis. Potential interventions directed against mucosal and cutaneous HPV, including both prophylactic and therapeutic approaches, are shown in Figure 1. Thus far, few compounds have been identified that inhibit HPV oncogenes and that might be considered as antivirals. Other therapeutic strategies targeting E6 and E7 oncoproteins include antisense RNA and DNA molecules [60], ribozymes targeted against HPV oncogene transcripts [61], and methods of silencing E6 and E7 gene expression [62,63]. However, all these approaches are still in an early development phase, and translation into clinical practice may be affected by safety issues.

More advanced approaches to inhibit HPV-induced disorders are represented by vaccination strategies. Currently, three vaccines consisting of L1-VLPs are licensed [64]. They are directed against HPV 16 and 18 (Cervarix), HPV 6, 11, 16, and 18 (Gardasil) and HPV 6, 11, 16, 18, 31, 33, 45, 52, and 58 (Gardasil-9), respectively, and efficiently prevent anogenital tumors caused by the targeted HPV types due to the induction of neutralizing antibodies [5,7,65]. These vaccines, however, are largely type-specific, providing no or low cross-protection against other HPV types, in particular beta HPVs [66], and show limited efficiency in immunosuppressed patients [27]. Regarding vaccination against beta HPVs, it must be considered that in contrast to alpha HPVs, no high-risk beta HPVs have been identified. Instead, a broad spectrum of beta HPVs is detected in AK and cSCC, with no specific types dominating, requiring coverage of a large number of different HPV types. As beta HPV infection occurs soon after birth, preventive vaccination must be very early in life. However, considering the potential beneficial effects of beta HPV infection, the prevention of beta HPV infection may be even disadvantageous. On the other hand, therapeutic vaccination to boost antiviral immunity may be more effective to prevent skin tumor formation, as it is directed against virus-infected cells in precancerous lesions.

### 4.1. Management of HPV-Associated Cutaneous Lesions with Licensed HPV Vaccines

Since the efficacy of VLP-based vaccines is based mainly on induction of neutralizing antibodies preventing primary infection, they were not recommended to treat prevalent HPV infection [67]. Treatment of existing HPV infection rather requires therapeutic vaccines that target E6 and E7 proteins, which are constantly expressed in infected cells. Nevertheless, L1-VLP vaccines have been used to manage different lesions with active or pre-existent HPV infection. Indeed, there are some reports on successful treatment of cutaneous warts and genital warts with HPV L1-VLPs [68,69].

For instance, Shin et al. reported 62.2% complete clearance of recalcitrant cutaneous warts after application of Gardasil-9 [70]. Other studies, however, showed a lack of efficacy of both quadrivalent and nonavalent vaccines in the majority of patients with verruca vulgaris [71]. Similarly, several studies reported on clinical responses of patients with anogenital warts treated with the bi-, quadri-, or nonavalent vaccine [72,73,74,75,76]. In a study comparing standard therapy (cryotherapy, nitrizinc complex, Imiquimod 5%, or PDT) and combined therapy (standard therapy plus vaccination with Gardasil-9) for treatment of anogenital and oral warts, clinical response (complete or partial clearance) was significantly higher in patients receiving combined therapy [72]. As with cutaneous warts, lack of efficiency of HPV vaccines in treatment of genital warts was also reported [77]. In a large study including >500 patients with genital warts receiving the quadrivalent HPV vaccine or placebo in addition to topical treatment (imiquimod or podophyllotoxin), no benefit of the HPV vaccine was demonstrated [78]. Whereas in a few studies no wart recurrence after successful treatment with HPV vaccines was observed [79], a large register-based study from Norway showed that administration of a quadrivalent HPV vaccine after the first manifestation of anogenital warts did not prevent subsequent disease episodes, indicating no protection of the vaccines against recurrent disease [80].

In addition, successful treatment of keratinocyte carcinomas using the quadrivalent and nonavalent Gardasil vaccine has also been reported [81,82]. By screening registry data of patients under immunosuppressive treatment for transplantation or autoimmune disease and patients with cancer and chemotherapy that had received three doses of Gardasil-9, a significant decrease in dermatologic procedures related to skin cancer was noticed in immunosuppressed patients after administration of the vaccine, indicating Gardasil-9 vaccination may decrease tumor burden in these patients [83]. Gardasil-9 was also applied in patients with AK. In a case series of 12 immunocompetent patients with high AK burden and insufficient response to previous conventional AK treatments, Gardasil-9 was administered at month 0, 2, and 6 in addition to conventional AK therapies. Twelve months after the beginning of vaccination, the total number of AK lesions was reduced by 85% on an average [84]. However, it is unclear to what extent Gardasil-9 vaccination contributed to the clinical response, as the case series did not include a control group receiving conventional therapy only.

Genital warts are caused mainly by HPV types 6 and 11, which were covered in the quadrivalent and nonavalent HPV vaccines. Although HPV infection is already established in genital warts, antibodies induced by vaccination may neutralize virus particles resulting from virus replication and prevent ongoing productive infection in the mucosal epithelium. In contrast, HPV types mostly associated with cutaneous warts, AK, and KC are not specifically targeted by the licensed HPV vaccines. The therapeutic effect in these cases may result from antigenic similarity of the L1 capsid proteins or, more likely, by nonspecific immune stimulation induced by the adjuvants in the vaccine formulations. Administration of the quadrivalent HPV vaccine in an immunosuppressed female patient with WILD syndrome (warts, immunodeficiency, lymphoedema, and anogenital dysplasia), presenting with both cutaneous and genital warts, resulted in regression of cutaneous warts but not genital warts. None of the HPV types covered by the vaccine was detected in cutaneous warts, whereas HPV 6 was found in the genital warts [85]. The clinical response of cutaneous warts only indicate that the adjuvant of the vaccine may be more important than the HPV specific constituents for the clinical response in lesions with active HPV infection.

### 4.2. Next Generation VLP-Based Vaccines for Beta HPV-Associated Skin Cancer

Due to the large number of different beta HPV types detected in skin cancers, with no particular type predominating, and considering that these cancers occur mainly in older and immunosuppressed patients, a vaccine with broad protecting activity is required that is effective in immunocompromised individuals and those already infected with beta HPVs.

Using the *Mastomys natalensis* PV (MnPV)/*Mastomys coucha* mouse model, Vinzón et al. showed that vaccination with L1-VLP-based vaccines against MnPV prevented formation of skin tumors in both healthy and immunocompromised animals [86]. Moreover, in animals already infected with MnPV, vaccination caused maintenance of a low viral load and prevented virus spread and tumor formation [86]. The study thus provided strong evidence that, in principle, vaccination should be feasible to prevent HPV-associated skin tumors in humans, even under immunosuppression. L1-VLPs, however, are largely type-specific, and thus impractical for use against the diverse group of beta HPVs found in human KC, as a sufficiently multivalent L1-VLP vaccine covering the more than 50 beta HPV types is technically difficult to realize.

In contrast to L1-VLPs, peptides derived from the minor capsid protein L2 or recombinantly expressed full length L2 were shown to generate a broad range of cross reactivity due to highly conserved regions among HPV types [87,88]. In a large-scale analysis of HPV16 L2 gene sequences from different geographic areas, the conservation of N-terminal L2 specific epitopes was confirmed [89]. No amino acid changes were detected in the region aa17-36 used for vaccination. A variation adjacent to that epitope (D43E) was identified; however, it is not known whether it affects the tertiary structure of the L2 protein. Furthermore, antibody titers induced by L2 antigens are significantly lower compared to L1-VLPs [90,91]. Several strategies have been utilized to improve immunogenicity of L2, as for instance the use of concatemers [88,92], fusion of L2 peptides to highly immunogenic compounds, like bacterial flagellin or thioredoxin [93,94,95], insertion of L2 peptides in heterologous virus capsids, like *adeno-associated virus* (AAV), *adenovirus* or bacteriophages [96,97,98,99], *Lactobacillus*-based recombinant vaccines [100], or chimeric vaccines consisting of the HPV L2 peptide RG1 (aa 17-36) in mucosal HPV L1-VLPs [101,102].

A vaccine of HPV16 RG1 inserted into the HPV16 L1 DE-surface loop, which contains the immunodominant neutralizing epitopes, induces cross-neutralizing antibodies to other mucosal HPVs and also some beta HPV types. The additional RG1 epitope did not affect HPV 16 L1-specific immune responses (high antibody titers and cytotoxic T cells to HPV 16) [103]. Thus, the vaccine combines HPV16-specific immunogenicity based on L1-VLP and broad-spectrum immunogenicity based on L2. Currently, the HPV16 VLP-HPV16 RG1 is produced under cGMP (current good manufacturing practice) and tested in a Phase I study as potential vaccine candidate (Pathovax LLC) [104].

In addition, the HPV16 VLP-HPV16 RG1 and another novel L2-based vaccine candidate (CUT-PANHPVAX) were recently investigated in the *M. coucha* natural infection model [105]. CUT-PANHPVAX represents a fusion protein of L2 peptides (aa20-38) from HPV types 1, 2, 3, 4, 14, 15, 22, 36, 41, 76, 88, and 95, respectively, inserted into the thioredoxin scaffold of *Pyrococcus furiosus* and fused to the OVX313 oligomerization domain (a hybrid derivative of the complement inhibitor C4-binding protein), which allows assembly to heat-stable heptamers [106]. Both vaccines induced robust cross-reactive and -protective antibodies against MnPV L2 that prevent development of MnPV-induced skin tumors [105]. Nevertheless, reactivity against L2 differs among both vaccines, as HPV16 RG1 VLP shows weaker cross neutralization of MnPV pseudovirions than CUT-PANHPVAX, indicating lesser cross-protection against other HPVs [105]. The limitation to induce broad cross-protection against other HPV types probably results from lower sequence identity of the RG1 epitope of mucosal and cutaneous HPVs. Indeed, when combining the RG1 epitope of HPV16 with beta HPV RG1 displayed within the L1 DE loop of HPV16 and HPV18, a better cross-neutralization potential for cutaneous HPV was achieved [101,107].

### 4.3. Therapeutic Vaccination Against Viral Oncoproteins

L1-VLP vaccines induce long-lasting high antibody titers and provide long-term protection, but they do not impact pre-existing HPV infection, probably because the basal keratinocytes harboring the viral infection do not express capsid antigens at detectable levels, and therefore are not attacked by vaccine-induced antibodies, whereas the infected cells in the upper epithelial layers that do express capsid antigens will be sloughed off shortly by desquamation. Thus, L1-VLPs essentially do not have therapeutic potential and are restricted to prophylaxis primarily. Therapeutic vaccines against HPVs usually target products of viral early genes, mainly the oncogenes, E6 and E7, which are obligatorily expressed in all HPV-infected cells [108]. Such vaccines are designed to activate HPV specific cytotoxic T cells and T-Helper cells to kill HPV-infected cells [109]. Vaccines based on E2 antigen are also of interest. As a negative regulator of E6 and E7, E2 expression in precancerous lesions is higher than E6 and E7. In addition, E2 is required for HPV replication, so that E2 vaccines may be used for the treatment of precancerous lesions and genital warts [110].

Different strategies have been used to develop therapeutic vaccines against HPV, including live vector-based vaccines (bacterial- or viral-based), peptide- and protein-based vaccines, nucleic acid-based vaccines, and whole cell vaccines [108]. In another approach, activation of innate immunity was shown to induce adaptive immune responses, preventing the development of skin cancer [111]. Using a HPV8 transgenic mouse model the treatment with poly (I:C) (polyinosinic-polycytidylic acid) which binds to melanoma differentiation-associated protein 5 (MDA5) and Toll-like receptor (TLR) 3, similar to viral nucleic acids, induces a T-cell response directed against viral antigen expressing cells in the skin. These T cells most likely represent tissue resident memory T cells and are required to prevent tumor formation. The technique corresponds to an autovaccination procedure with the advantage of activating adaptive immunity against beta HPV types that are present in the skin, but do not need to be specified.

Whole cell vaccines include dendritic cells (DCs) obtained from patients that are in vitro stimulated with HPV antigens or transfected with vectors expressing HPV antigens, then transferred back to patients. The safety and immunogenicity of DCs pulsed with HPV16/18 E7 antigen have been shown in patients with cervical cancer [112]. However, DC-based vaccines are difficult to produce on a large scale and different processing methods may affect vaccine quality.

Vector-based vaccines include attenuated bacteria, like *Listeria monocytogenes* or replication-deficient viruses, like AAV, *adenovirus*, or *vaccinia virus*, that carry genes for HPV-specific antigens to replicate in host cells and induce an immune response against HPV. Despite inducing strong immune responses and promising results in some phase I/II studies, the use of these vaccines may be limited by safety issues, in particular in immunocompromised patients [113].

In contrast, protein/peptide and DNA vaccines are generally safer, but on the other hand are less immunogenic, requiring adjuvants or immunostimulatory molecules to increase immunogenicity. Joined peptides of HPV E6 and E7, covering the complete sequence of both oncogenes and emulsified with the adjuvant Montanide ISA-51, were used to treat HPV16 positive cancers together with PD1 inhibitor nivolumab and resulted in a higher response rate compared to nivolumab alone [114]. The vaccine alone, however, did not affect regression of cervical cancer, probably because vaccine-activated T cells are inhibited by an immunosuppressive tumor microenvironment [115]. In another study, heat-shock protein (HSP) 110 was used as an adjuvant to enhance the immune response of cytotoxic T cells (CTL) directed against HPV16 E7 epitopes. In a mouse model, immunized animals induced a strong CTL response and inhibited the growth of established tumors [116].

Immunogenicity was also significantly improved by fusing HPV16 and HPV18 full length E7 to the TLR4 agonist fibronectin extra domain A (EDA) to direct the antigen to dendritic cells. In combination with TLR3 agonists Poly (I:C) and Poly-ICLC, the recombinant E7-EDA protein induced E7-specific CTL responses and eliminated established genital or subcutaneous HPV16 tumors in mice with high efficiency [117].

Like L1-VLPs, L2-based vaccines also do not have therapeutic potential, but they may induce both prophylactic and therapeutic immunity when fused with the HPV oncoproteins E6 and E7. Such fusion proteins might be particularly useful for vaccination against beta HPVs to which most patients of interest have already been exposed, as beta HPVs are acquired early in childhood [118,119]. An example for this strategy is represented by TA-CIN (tissue antigen–cervical intraepithelial neoplasia), a fusion protein of HPV16 L2, E6 and E7, which is able induce antibodies and T-cell responses and was shown to provide both prophylactic and therapeutic activity in a pre-clinical mouse model [120]. The immunogenicity of TA-CIN was subsequently verified in clinical studies and confirmed induction of humoral and cellular immune responses in humans [121,122].

In a similar approach, a fusion protein of HPV6 E7 and L2 was used to treat HPV6 caused genital warts. Induction of humoral and T-cell responses against L2 and E7, respectively, was shown in clinical studies [123,124]. However, therapeutic vaccination failed to increase the efficacy of conventional therapies in a study of 457 subjects with anogenital warts that received the vaccine or placebo, in combination with ablative therapy or podophyllotoxin, possibly due to the frequent spontaneous regression of genital warts and the large proportion with multiple HPV types [125].

DNA vaccines usually represent recombinant bacterial plasmids encoding the antigens of interest under control of high-efficiency eukaryotic promoters. After injection DNA vaccines must enter the nucleus of eucaryotic cells to induce expression of antigens, which are then presented by MHC class I molecules to activate the immune system. The inability of the DNA to amplify by itself results in poor immunogenicity of DNA vaccines and requires the use of strong adjuvants or immunostimulatory substances. Vaccination of mice with a DNA vaccine composed of HPV16 L2, E6, and E7 linked to calreticulin (CRT) was shown to induce E6- and E7-specific CD8+ T cells that conferred significant anti-tumor effects against E6/E7-expressing tumors and an L2-specific protective antibody response against HPV16 was demonstrated [126]. The vaccination approach was also tested in the MmuPV1 model using hCRT-MmuPV1-E6-E7-L2 and induced CTL responses against E6 and E7 and anti L2-antibodies [127]. Whereas the vaccine causes disappearance of persistent papillomas in treated mice, it is unclear whether it confers protection against formation of tumors associated with human PV types.

Another vaccine strategy based on nucleic acids is represented by mRNA vaccines. Compared to DNA vaccines, self-amplifying mRNA vaccines have the capacity for autonomous replication, and as ligands of TLR7 and TLR8, they may be considered natural adjuvants that can induce strong immune responses [128,129]. Thus far, mRNA vaccines are not well studied to protect against HPV infections and diseases. However, with the outbreak of the new coronavirus in 2020, these vaccines experienced significant improvements regarding safety and efficiency, and therefore mRNA vaccines are likely to be developed for HPV in the future.

## 5. Concluding Remarks

Many of the described candidates for therapeutic vaccination of HPV were studied in clinical phase I, II, and III trials. Although they were successful in animal models, there are concerns of limited clinical efficiency in human HPV-associated tumors. Indeed, it is important to consider that immunosuppressive microenvironments of human tumors, characterized by immunosuppressive cytokines and immunosuppressive cells like M2 macrophages, myeloid derived suppressor cells (MDSC), or cancer-associated fibroblasts (CAF), may affect the efficacy of vaccine-induced T cells [130]. Therefore, it is a primary goal of cancer therapy to reverse the immunosuppressive tumor-promoting environment into an immune-activating, tumor-destroying milieu. In addition, immunogenicity of HPV vaccines needs to be improved further. A promising strategy is represented by mRNA vaccines, encapsulated in cationic lipid nanoparticles, which are highly immunogenic due to their mRNA content and the use of lipid nanoparticles, both representing natural adjuvants. The combination of therapeutic vaccination with immune checkpoint inhibitors to reverse immunosuppression of cytotoxic T cells is another strategy [131]. Indeed, anti PD-1 antibodies pembrolizumab and nivolumab, have shown some effectiveness in combination with HPV vaccines [132]. However, in contrast to the three licensed VLP-based prophylactic vaccines against alpha HPVs, none of the vaccine candidates for beta HPVs have thus far been approved for application in humans.

## Data Availability

No new data were created or analyzed in this study.
